# “It’s because We are ‘Loose Girls’ That’s why We had Children with MINUSTAH Soldiers”: A Qualitative Analysis of Stigma Experienced by Peacekeeper-Fathered Children and Their Mothers in Haiti

**DOI:** 10.1177/08862605211072178

**Published:** 2022-02-22

**Authors:** Luissa Vahedi, Heather Stuart, Stéphanie Etienne, Sandra Wisner, Sabine Lee, Susan Andrea Bartels

**Affiliations:** 1 Department of Public Health Sciences, 4257 Queen’s University, Kingston, ON, Canada; 2 Komisyon Fanm Viktim Pou Viktim (KOFAVIV), Port-au-Prince, Haiti; 3 Institute for Justice and Democracy in Haiti, Boston, MA, USA; 4 Department of History, 1724 University of Birmingham, Birmingham, UK; 5 Department of Emergency Medicine, 4257 Queen’s University, Kingston ON, Canada

**Keywords:** Haiti, Peacekeeping, United Nations, Sexual Violence, Stigma, Peace baby, Sexual Exploitation, Sexual Abuse

## Abstract

Sexual abuse and exploitation (SEA) perpetrated by UN peacekeepers while on mission is a violation of human rights and undermines the goal of upholding human rights in countries that host peacekeeping missions. In addition to survivors, children fathered by peacekeepers are also victims of SEA that need protection. Stigma poses negative life course consequences for SEA survivors and their peacekeeper-fathered children. However, there is a considerable lack of empirical research concerning the stigma experiences of SEA survivors and their children in post-colonial contexts. The present study addresses this knowledge gap by drawing on The United Nations Stabilization Mission in Haiti as a case study to examine the lived experiences of stigma among SEA survivors and their resultant children. Using 18 qualitative semi-structured interviews conducted in 2017 with Haitian women raising peacekeeper-fathered children, we organized qualitative codes according to Link and Phelan’s conceptual model of stigma. The stigmatization process was explored through the themes of labeling, stereotyping, separation, and status loss and discrimination, as described by Link and Phelan. In addition, we nuanced the lived experiences of stigma by discussing the buffering roles of familial acceptance, skin phenotype, and the Haitian context. The findings have implications for the UN. We advocate that stigma be recognized and acted upon as a fundamental protection concern for SEA survivors and their children. Accordingly, the UN has an obligation to provide stigma-related supports for victims and complainants as well as to facilitate long-term child support for the children left behind by peacekeepers.

## Introduction and Background

Sexual exploitation and abuse (SEA) perpetrated by UN peacekeepers is one form of gender-based violence that undermines the goals of conflict resolution, political stability, and human rights within countries that host peacekeeping operations (POs). Nearly all POs have been associated with some degree of SEA ever since reports of peacekeeper-perpetrated SEA emerged publicly in the 1990s (Nordås & Rustad, 2013; United Nations Peacekeeping, 2021; United Nations Secretary General, 2017). Notably, POs in the Democratic Republic of the Congo (DRC), Liberia, Central African Republic, Sierra Leone, South Sudan, and Haiti reported among the greatest number of SEA allegations (United Nations, 2021).

Scholars have investigated factors that are positively associated with reported SEA, namely, the mission’s mandate, disciplinary breakdown of contingents, number of deployed personnel, a previously established economy of transactional sex, elevated violence during conflict, and low host country GDP per capita are positively associated with reported SEA (Beber et al., 2017; Moncrief, 2017; Neudorfer, 2015; Nordås & Rustad, 2013). On account of the reliance on transactional sex as a source of income, large mission size, extreme poverty, as well as organized crime and civilian violence, MINUSTAH and Haiti were at an elevated risk for peacekeeper-perpetrated SEA (Heinemann, 2006; Logie, 2017; Perito & Robert, 2008). Moreover, publicized media reports revealed that MINUSTAH peacekeepers were implicated in sexual misconduct including, sexual abuse, sex trafficking, and the exploitation of children (Al Jazeera, n.d.; The Washington Post, 2017; Williams, 2007).

One enduring consequence of peacekeeper-perpetrated SEA is pregnancy; local women from a variety of peacekeeping contexts have reported resultant pregnancies (Simić & O’Brien, 2014; United Nations, 2021; Vahedi, Stuart, et al., 2021). Across POs between 2010 and 2020, 345 paternity claims involving peacekeepers have been reported to the UN, 37 of which occurred during MINUSTAH (United Nations Peacekeeping, 2021). While peacekeeper-fathered children have been referenced in UN documents and scholarship as also being victims of SEA in need of support (Simić & O’Brien, 2014; United Nations, 2008, 2019; Vahedi et al., 2020; Wagner et al., 2020), little is empirically known of their lived experiences and the challenges they face.

Peacekeeper-fathered children are one group of Children Born of War (CBOW): children with one parent who is a local civilian (most often the mother) and another parent who is a member of a foreign army, rebel group, or peacekeeping force (usually the father) (Mochmann, 2017). Although MINUSTAH was established in response to organized crime and civil unrest, rather than armed interstate conflict, peacekeeper-fathered children in Haiti are considered CBOW; MINUSTAH peacekeepers included military contingents of nation states and were authorized to use up to and including deadly force during mission (United Nations Department of Peace Operations, 2020; Wills, 2018). Previous scholarship has illustrated that CBOW face stigmatization, discrimination, child maltreatment, and adverse identity formation due to their unique biological origins and paternal identity (Daniel-Wrabetz, 2007; Kaiser et al., 2015; Mitreuter et al., 2019; Vahedi et al., 2020; Wagner et al., 2020; Wagner et al. forthcoming).

Although scholars have postulated that peacekeeper-fathered children and their mothers face distinct experiences of stigma (Simić & O’Brien, 2014; Wagner et al., 2020), literature that comprehensively details the stigma experienced by peacekeeper-fathered children and their mothers is in its infancy. One recent study of mothers and peacekeeper-fathered children in the DRC illustrated the bi-directional and transgenerational stigma experienced by the mother and child within the wider context of poverty (Wagner et al., 2020). The mothers’ extramarital relations, connection to the UN, and single parenting negatively impacted the peacekeeper-fathered children through the corresponding conditions of perceived illegitimacy at birth, mixed ethnicity, and not having a father (Wagner et al., 2020).

To build upon the current discourse surrounding stigma experienced by peacekeeper-fathered children and their mothers, we draw on an existing theoretical model of stigma. Link and Phelan’s (2001) conceptualization of stigma describes the process through which social identities are linked to undesirable stereotypes that ultimately result in reduced life chances. As defined by Link and Phelan, “stigma exists when elements of (i) labeling, (ii) stereotyping, (iii) separation, and (iv) status loss and discrimination occur together in a power situation that allows them” (2001, p. 377). Thus, stigmatization encompasses four interrelated elements: the *labeling* of human differences, ascribing negative *stereotypes* to the labels, placing persons in distinct stereotype tropes *that separate “us” from “them,”* and the culminating experience of social/structural inequality, such as unequal outcomes, disapproval, rejection, and exclusion, due to *discrimination and status loss* (Link & Phelan, 2001). Link and Phelan’s (2001) conceptualization of stigma draws attention to power hierarchies. The persons enacting stigma occupy positions of greater power compared to the stigmatized: this power gradient plays a central role in enacting the resultant status loss and discrimination experienced by the persons being stigmatized, as modulated through the processes of labeling, stereotyping, and separation.

Link and Phelan’s conceptualization is the only model that fully integrates structural power dynamics and subsequent discrimination within the process of stigmatization, thereby offering a robust and holistic understanding of stigma that extends beyond the individual into social and structural inequalities (Yang et al., 2007). While stigma is a shared human phenomenon, a single formulaic experience does not adequately capture contextual nuances. Across countries, cultures, and histories different meanings, practices, and social structures diverge the experience of stigma (Yang et al., 2007). Despite this, the majority of stigma research has focused on high income countries. Consequently, little is known about the generalizability or transferability of Link and Phelan’s (2001) theoretical model to humanitarian settings and in relation to women and girls who are survivors of SEA.

### Purpose

This research utilizes The United Nations Stabilization Mission in Haiti (MINUSTAH) as a case study to examine the lived experiences of stigma among SEA survivors and their resultant children. Drawing on the perspectives and lived experiences of 18 Haitian mothers of peacekeeper-fathered children, we employ Link and Phelan’s (2001) conceptualization of stigma to describe the stigmatization experienced by the mothers, who are survivors of SEA, and their peacekeeper-fathered children. In doing so we conceptualize stigma as an essential protection concern and a policy-actionable process through which SEA survivors and peacekeeper-fathered children face considerable life challenges, particularly with respect to reduced socio-economic chances.

## Methods

Qualitative interviews with 18 Haitian women raising peacekeeper-fathered children were conducted between October and December 2017. These interviews followed a larger mixed-methods study conducted earlier in 2017 to examine broader community perceptions about the experiences of women and girls living in communities that hosted MINUSTAH bases (Lee & Bartels, 2019; Vahedi et al., 2021c). 

The current study was implemented in collaboration with The Komisyon Fanm Viktim pou Viktim (KOFAVIV): a grassroots community-based organization in Haiti that provides services and support to survivors of sexual violence, and Bureau des Avocates Internationaux (BAI): Human rights lawyers and advocates from Haiti and the US who represent Haitian SEA survivors. The authors collaborated with KOFAVIV to create the semi-structured interview guide, conduct research training, develop a recruitment strategy, and implement the research. The survey was created in English, translated to Haitian Kreyòl, and then back translated to English to confirm accuracy. KOFAVIV team members guided implementation decisions such as choice of recruitment sites and provided valuable input on Haitian culture and the local context. Interviews were conducted by five female KOFAVIV research assistants who had prior experience working with survivors of sexual violence in Haiti. All research assistants underwent a 4-day training (led by SAB), which included: recruiting research participants, research ethics including informed consent, semi-structured interviewing, audio recording interviews, as well as providing psychological support to participants and referral for services if needed.

Any female participant who shared a first-person narrative about having a peacekeeper-fathered child in the earlier 2017 mixed-methods study was invited to participate in the follow-up qualitative interviews, which are presented here. From this purposeful sample, other women in the community raising peacekeeper-fathered children were recruited using a snowball technique. Haitian women/girls who were 16 years or older and raising peacekeeper-fathered children were eligible for inclusion. Semi-structured interviews were conducted in Port-au-Prince (including around UN bases at Cité Soleil, Tabarre and Log Base), Hinche, Leogane, Port Salut, Port-du-Paix, Cap Haitien, Fort Liberté, and Saint Marc. Each participant completed a single interview in Haitian Kreyòl in a private location such as the participant home or at the office of a local NGO. All interviews were audio recorded in Kreyòl and then transcribed and professionally translated to English.

Verbal informed consent to participate was given by participants after having the opportunity to ask questions about the research. As a token of appreciation, $1 USD of mobile phone credit was provided to each participant. Additionally, light refreshments were made available during the interview and any incurred travel-related costs to participate were reimbursed (generally about $1 USD for local mini-buses). The Queen’s University Health Sciences and Affiliated Teaching Hospitals Research Ethics Board approved the study protocol (number #6021205).

We employed a phenomenological approach to examine the semi-structured interview transcripts, which probed participants on personal experiences of stigma within the family and community. Using phenomenology, we sought to create an explanation of stigma based on the descriptions, perceptions, experiences, and feelings of Haitian SEA survivors who are also mothers to peacekeeper-fathered children. Thus, phenomenology guided data analysis, enabling the research team to reduce individual participants’ experiences into the overarching essence of how stigma was experienced by the SEA survivors and their children (Aspers, 2009; Moustakas, 1994).

All transcripts were coded by LV who iteratively analyzed each transcript for meaning units and organized them in NVIVO 12.2.0. After initial coding, transcripts were re-read for further refinement into more precise codes that represented unique aspects of stigma-lived experiences. A combination of data-driven codes emerging from the raw data as well as theory-driven codes derived from Link and Phelan’s (2001) conceptualization of stigma were developed. LV consulted with SAB and HS regularly to discuss the various codebook drafts and explore emerging themes. The codes were critically assessed to ensure that they were contextually accurate and represented distinct meanings. A final read of the transcripts helped to develop a framework that illustrated the lived experiences of stigma among the SEA survivors raising peacekeeper-fathered children. KOFAVIV and BAI team members were consulted to identify any misperceptions in the coding as well as to incorporate Haitian culture and values into the framework.

Research group meetings established a reflexive research practice that focused on the authors’ critical self-reflection. As such, reflexivity was paramount to establishing the credibility of the analysis and findings (Tracy, 2010). Furthermore, SAB and LV traveled to Haiti in May 2019 to contextualize the qualitative findings, hold discussions with KOFAVIV team members, and to engage in reflexive journaling.

## Results

### Study Sample

Seventeen participants were biological mothers of peacekeeper-fathered children, and one participant was the maternal grandmother of the child. When the interviews were conducted in late 2017, all participants were aged between 20 and 42 and the peacekeeper-fathered children ranged in age from 8 months to 12 years. At conception, the youngest reported maternal age was 14 and the oldest reported maternal age was 29. Given the legal age for sexual consent in Haiti is 18 years old, SEA involving adolescents under the age of 18 is considered statutory rape under Haitian law (Age of Consent, 2021). Thus, our sample consists of sexual relations involving peacekeepers and Haitian women and girls that can be classified as statutory rape in addition to exploitative transactional sex, sexual abuse, and longer-term transactional sex relations imbedded in perceptions of “love” (Vahedi et al., 2019). The average number of children per participant was about 2, including the peacekeeper-fathered children. About one third of participants could identify the peacekeeper father by name or nationality. When interviewed, participants worked as street vendors, factory workers, teachers, sex workers, or domestic maids. There was also one case reported by the child’s maternal grandmother, of the mother having immigrated to Chile for employment purposes. Unemployed participants relied on familial support to sustain their needs. In our data, all women and girls were eventually abandoned by the peacekeeper father who returned back to his country of origin either at the end of his term of duty or due to dismissal (Vahedi et al., 2020).

### Labeling

The process of stigmatization begins with labeling biological and social human differences in a manner that is socially relevant and visible (Link & Phelan, 2001). Labeling brings forward select human attributes while rendering others as invisible and less socially salient. In the case of peacekeeper-fathered children in Haiti, this labeling manifested as community members and peers coining phrases that brought attention to the paternal origin of peacekeeper-fathered children, such as “child of the MINUSTAH” or more simply “MINUSTAH.” Such socially ascribed labels were often connected to unpleasant consequences, such as the mothers feeling uncomfortable and experiencing discrimination, and hence were perceived negatively. Ultimately, “Child of the MINUSTAH” connected the peacekeeper-fathered children to Haiti’s legacy of peacekeeping and foreign intervention:

You know since the MINUSTAH left it felt uncomfortable and also every time someone sees you [and] they say, ‘oh this is the child of the MINUSTAH,’ that made me uncomfortable (Hinche).

Another participant goes on to further explain that the MINUSTAH label distinguished the peacekeeper-fathered child from others. Being labeled a “child of MINUSTAH” also foregrounded the father’s abandonment and highlighted the child’s foreign ancestry as well as connection to UN peacekeeping and foreign intervention, while rendering less visible the child’s need for protection and support:

She knows that he is not Haitian and is a MINUSTAH [personnel], as most people always called them. Even the children the same age as her would call her like that, discriminating against her in that way (Port de Paix).

Similarly, another participant described peer-to-peer labeling:

Once the child is born other children will make sure they let him know he is the child of a MINUSTAH (Hinche).

While the children were marked as “child of the MINUSTAH” by their peers and community members, the mothers’ labeling experiences related to their perceived sexuality. The label of “loose girls” (in Kreyòl “pa bon”, which is used colloquially in Haiti to refer to women or girls who have multiple sex partners) highlighted the mothers’ supposed role in “willfully” participating or seeking out sexual relations with MINUSTAH peacekeepers. At the same time, the label “loose girls” obscured the mothers’ experiences as SEA survivors and in some cases, victims of statutory sexual assault:

People used to say it is because we are “loose girls” that is why we had children with MINUSTAH soldiers (Port Salut 4).

Among some participants, the victim-blaming label intensified: the respondents were perceived as deliberately and willfully planning to conceive children with “white” MINUSTAH peacekeepers. In such cases, the label was used to justify the adverse living conditions experienced by the single Haitian mothers in that “loose girls” were perceived as having purposefully conceived mixed-race children with MINUSTAH peacekeepers:

They sometimes talk, and say I went and made a white man get me pregnant and so look at me now, my living conditions are bad. (Port Sault 2)

### Stereotyping

The second component of stigma involves stereotyping. According to Link and Phelan (2001), the undesirable characteristics that are associated with a label form the stereotype. The process of associating labels with negative stereotypes occurs automatically and is unchallenged; the stereotype is simply accepted as the natural state of affairs. The labels “loose girl” and “child of the MINUSTAH” are linked to the undesirable characteristics: mothers’ promiscuity, paternal abandonment, and subsequent poverty.

The participants described that community members associated the mothers with promiscuity, particularly because women and girls seemingly desired sexual relations with “white” MINUSTAH peacekeepers and were active agents in engaging in sexual relations with peacekeepers:

But you know neighbors, they would say that “this girl is promiscuous, she had a child with the white men, she slept with the white men” (Tabarre 3).

In addition to the stereotype of promiscuity, some participants also identified that community members differentiated between levels of promiscuousness according to whether children were conceived as a result of sexual interactions with peacekeepers. That is, community members stereotyped the sexual behavior of women and girls who conceived children with MINUSTAH peacekeepers as being less acceptable compared to the behavior of women and girls who engaged in sexual relations that did not result in pregnancy. In such cases, the pregnancy and child were visible markers of SEA:

Since we have children, they see our [behavior] as ‘uglier’ [more unacceptable than theirs] than others who used to do the same thing, but since they don’t have children, they [can act as if] they were never part of these things. It bothered me because we weren’t the only ones [having sexual relations] with the MINUSTAH [peacekeepers], there were many other people at the MINUSTAH’s base (Port Salut 4).

The mother’s assumed promiscuity and perceived socially non-conforming sexual behavior were transferred to the peacekeeper-fathered child. Stereotypes related to child poverty and adverse living conditions were linked to the mother–child relationship. For example, one participant described how the stereotype of the “promiscuous” mother (the term used in Kreyòl was pèlen which refers to women/girls with multiple sex partners) was implicitly associated with undesirable living conditions that affected both the mother and the child:

People in the neighborhood, the neighbors, they gossip. They talk a lot, some of them ask the child where their father is … Some people talk and say, well, you went and got pregnant by a MINUSTAH [peacekeeper], you are going to be eating shit with this child … I suffer a lot of setbacks with the child. They sometimes humiliate me, say a lot of other things that I can’t repeat. (Port Salut 2)

Given none of the peacekeeper fathers in our sample supported the mothers/children after delivery, the impoverished and promiscuous mother stereotype was also embedded within the context of fatherlessness and abandonment. Some participants contrasted their previous life aspirations against the difficult reality of single motherhood within the context of abandonment and limited or non-existent support from the UN and the peacekeeper father. In some cases, bearing the child of a foreign peacekeeper was initially seen as advantageous: a mechanism through which upward social mobility could be achieved in a setting where there are limited opportunities for women and girls. For example, one participant noted that the peacekeeper father’s abandonment precipitated hardships for the mother and child that juxtaposed her perception of “success”:

I thought that this child was my key to success in the future. But unfortunately, the reason I see that I have no success in my future is because he left, and I never heard from him. But he left me the child, as my reward (Saint Marc 3).

The peacekeeper-fathered child was stereotyped as a child whose UN-affiliated father abandoned paternal responsibilities. Thus, “the child of the MINISTAH” is a child who grows up in the absence of their biological father to face the realities of poverty and hardship:

He will never grow up normal at all because wherever he goes he will have the label ‘here is the child of the MINUSTAH,’ ‘here is the child of the MINUSTAH who did not take care of him,’ and ‘here is the child of the MINUSTAH who did not take care of him.’ (Hinche)

### Separation of “us” versus “them”

The third component of stigma describes the separation of persons who enact stigma from those who experience stigma (Link & Phelan, 2001). The unchallenged association of labels with undesirable stereotypes underscores the view that stigmatized persons are fundamentally different from other persons, thereby establishing a power hierarchy. Perceptions of fundamental differences rationalize the harmful treatment of “them” and perpetuate the belief that “they” are less human and less worthy than “us.” In our data, the separation of “us” versus “them” occurred at two distinct levels in relation to the mother and the child.

Based on their presumed agency in conceiving a child with a foreign peacekeeper as opposed to a Haitian man, women/girls who conceived peacekeeper-fathered children were distinguished from other women in the community (including those women/girls who experienced SEA but did not bear children). For example, one participant referenced that community members differentiated her from other Haitian women/girls based on her decision to “choose” the peacekeeper as a sexual partner to bear children with:

They say look at who you got pregnant for, you didn’t choose people from your own country (Port Salut 2)

Another participant explained that when knowledge of her pregnancy became public, friends who also experienced SEA but did not conceive children with peacekeepers distanced themselves from her. In essence, the conception and birth of a peacekeeper-fathered child created inconsolable differences between mothers of peacekeeper-fathered children and women and girls who were involved sexually with MINUSTAH peacekeepers but did not conceive children. Differences were rooted in more severe stereotypes associated with the perceived socially unacceptable sexual behavior and decision making among Haitian women and girls who became pregnant:

I know of some women who were in relationships with Africans, but they don’t have children with them. During the time that I was with the MINUSTAH [personnel] I had many girlfriends who were also with Africans. Around the same time when I got pregnant and had the baby all these friends abandoned me, they humiliated me and I never see them again. I never see them to this day. (Saint Marc 3).

In some cases, Haitian men and boys also perpetuated further differentiation between mothers of peacekeeper-fathered children and other women and girls within the community. In such cases, selectively “choosing” UN peacekeepers as romantic partners, was used in retaliation against the unreciprocated sexual advances of Haitian men/boys:

When I am walking by in the streets, if some young guys called me and I did not respond to them, they would try to talk down at me, always saying [it’s because] they were not MINUSTAH. That I had a child with a MINUSTAH. They would say things like that, and it would hurt me when I hear those words because I simply slept with someone in the MINUSTAH (Port de Paix).

The same participant further explained that other Haitian women/girls without children expressed that they would never “choose” to bear children from MINUSTAH peacekeepers over Haitian men. This sentiment was rooted in the community perception that their partners (presumably Haitian) would be less likely to abandon their biological children in the same way the foreign peacekeepers did or could be more easily contacted to obtain support, financially, or otherwise:

The same thing would happen when I got into an argument with some young ladies who haven’t had any children yet. They always say that they did not have children with MINUSTAH. If they had a child, the father would be there to take care of him/her. And a lot of other insults they would throw at me. Those insults troubled me but I would just accept everything (Port de Paix)

Moreover, when one participant was asked how her family reacted towards her peacekeeper-fathered child, she noted being blamed for the child’s hardships and was subsequently discouraged from seeking other romantic relationships with Haitian men:

My family’s reaction, they are always talking to me, blaming me. They are always reproaching me because the child is suffering. They are always reproaching me, telling me to stay alone and never have another partner because of all the struggles that I am having to go through with the child … Because the way the father of the child used to treat me, they never thought that he would do something like that to me. They are always preaching me, telling me not to go look for someone else anymore. But I am still living … always crying, tears in my eyes. [teary voice] I am humiliated a lot, a whole lot. (Saint Marc 3).

Due to the mothers’ perceived promiscuity in “choosing” to have sexual relations with peacekeepers, resultant children were seen as fatherless once their foreign fathers fled their paternal responsibilities in Haiti. The peacekeeper fathers, who occupy a privileged socio-economic position within the context of post-colonial Haiti, have the means to support the mother and child but abandoned their legal and moral responsibilities by leaving Haiti and were subsequently absolved due to impunity. Consequently, peacekeeper-fathered children were perceived as being “inferior” compared to Haitian children. For example, one participant stated:

People are calling my son left and right, telling him that he is to be inferior in comparison to other children who have both parents that are living in Haiti. (Tabarre 3)

### Status Loss and Discrimination

Labeling, stereotyping, and the separation of “us” versus “them” ultimately culminate in the final phase of stigmatization: status loss and discrimination (Link & Phelan, 2001). The process through which persons are labeled, automatically associated with harmful stereotypes, and set apart both overtly and covertly form an unequal social order whereby stigmatized persons face substantial adversity. Discrimination and loss of social status within the community were exemplified through participants’ experiences of public humiliation when with their child. In the following example, the participant specified that she was humiliated and insulted when she was out in public with the child, thereby attributing her experiences of stigma to the child and implying that her social status had changed as a result of the child’s presence in her life. In order to protect the child from discrimination, some participants relied on extended family members to relocate the peacekeeper-fathered child:

They made fun of me, humiliated me, they insulted me … Since I was being humiliated and insulted when [the child] was with me, I had to take him and left him with my aunt. (Cite Soleil 2)

The source of humiliation felt by the participants stemmed from the perceived “choice” of engaging sexually with peacekeepers. In our sample, sexual relations with peacekeepers and bearing children did not result in upward social mobility; rather, the peacekeeper father who had the means to provide for his child, evaded paternal responsibility, leaving the mother and child in a precarious situation economically and socially. Ultimately, the mothers of peacekeeper-fathered children were ‘blamed’ for the abandonment. For example, one participant expressed:

My family’s reaction...since they didn’t approve of my relationship with the MINUSTAH [officer]. They abandoned me, and kicked me out [of the house] to the point where even the community where I live was against me (Tabarre 4).

When the same participant was asked who helps her and her child financially, the same participant responded:

At this point, no one [specific] is helping me … My parents, since they saw me having the baby for this foreign nation, and he doesn’t take care of the child, they used to help me but they stopped, and abandoned me, they don’t help me anymore (Tabarre 4).

Stated simply by one participant in relation to her family and the community: *“They humiliated me because the MINUSTAH had left. (Hinche)”*

Another participant expressed that she was living a “miserable” life with her child due to the humiliation:

I am humiliated a lot – because the person you don’t expect to humiliate you is the one humiliating you. I don’t live well … I am living a miserable life with the child … No one is looking our way, there is no one to help me with the child (Saint Marc 3).

The participant then went on to describe a specific instance of discrimination enacted by one of her child’s peers. Due to the closeness of mother–child relationship, discrimination experienced by the peacekeeper-fathered child also affected the mother, who described also being negatively affected by the experience:

My child’s name is [x] … while the neighbor’s child was drinking his juice, he asked, [x], would you like some? [x] said yes, he spat in the juice and then gave it to my child. That really bothered me. That hurt me. I spent a week worrying about that. When I think of the way [x]’s father used to treat me, brought us juice, candies. In my house, we couldn’t get enough of these things. We didn’t have enough space to put them. And the way we were with all the kids in the area, and they knew that too – and then to see that it was the neighbor’s child who spit in the juice to give it to my child. I worried about that a lot. That hurt because I said, if I had enough to give my child what he needed, he wouldn’t need to ask other people’s children. (Saint Marc 3)

In some cases, cumulative exposure to discrimination such as public humiliation and status loss such as being kicked out of the house or devalued resulted in suicidal ideation:

Those humiliations affected me, I wasn’t always able to cope. Sometimes, I felt like I wanted to kill myself because I have never thought that I would be in this position with my life. It bothered me. (Tabarre)

The mother expressed concern that the accumulation of discrimination enacted by peers and community members has the potential to negatively impact the child’s capacity to learn in an academic setting. For instance, in response to experiences of discrimination one participant stated:

That can impact his learning ability and when the child becomes older that can make it uncomfortable for him to stay in the area. (Hinche)

Further, discrimination experienced by both the mother and the child resulted in the avoidance of help seeking behaviors within the community. When asked about whether they sought assistance and financial support for child rearing responsibilities or were aware of the UN’s policy for SEA victims seeking assistance, participants described feeling ashamed to inquire

No, I haven’t looked because I feel easily ashamed. I wouldn’t like to go ask someone for them to not have what I ask or they insult me, I am forced to stay in silence. I don’t ask. (Saint Marc 3)

Similarly, another participant explained that the tight knit social dynamics of her small town of Port de Paix negatively affected her ability to seek support. Thus, the fear of being discriminated against while seeking help inhibited the accrual of resources and support:

I sit without looking because you know Port-de-Paix is a small city that is not as developed as to the other ones; because of any small search in the neighborhood and the entire city will be aware of everything that is going. In fact, it will become an insult to your person. You will be unable to walk around. So, I just kept my pain inside of me, just like that. (Port de Paix)

Reduced help seeking behavior and resource utilization reflects not only how mothers of peacekeeper-fathered children were devalued in public spaces but also how sigma enacted by others leads to internalized stigma, wherein participants feel shame for seeking help (Kennedy & Prock, 2018).

### The buffering role of familial acceptance, skin phenotype, and the social environment

While a majority of respondents spoke to components of Link and Phelan’s (Link & Phelan, 2001) conceptualization of stigma, there were also notable deviations. Stigma was not experienced uniformly among the participants. In particular, stigmatization differed according to the degree of acceptance from family and Haitian partners. In some cases, the child’s lighter skin phenotype was related to greater familial acceptance; however, such acceptance did not eliminate stigma enacted by members of the community more broadly.

The family units of some participants gradually accepted the peacekeeper-fathered child, acting as a buffer to the eventual status loss and discrimination, at least in the private sphere of the home. Thus, familial acceptance challenged the negative stereotypes associated with being fatherless and born to a woman/girl who “willfully” engaged in sexual relations with peacekeepers:

Now, my family really love him – but what they used to say before like oh, he doesn’t even have a father – but now they don’t judge on that anymore. They still love him. (Saint Marc)

Similarly, another participant described how experiences of stigma enacted by family members and the wider community lessened—although did not completely disappear—with the passage of time:

Things got so difficult between my family and I that I had to stay over some friends for weeks or months [at times]. Then, I would come back to the house whenever things calmed down again … Well, initially, there were problems, but for the time being, there’s no problem. Well, as the wrong has already been done, whether I take it in a good or in a bad way, that wouldn’t make any difference. Even though other people still see it as something wrong, but the situation is not as bad as it was. (Tabarre)

Other women reported they lived harmoniously in blended families with Haitian partners:

I told the man I was living with that I had the child with a MINUSTAH but as for the people in the community, I didn’t care about them … I am living with my partner and the child that I gave birth to, and another child for the man I’m living with. He never mentioned that to me, so I never get into any dispute with him (Tabarre 2).

However, living harmoniously in a blended family did not always equate with the elimination of social stigma enacted by community members. The same participant went on to further explain that the community would perceive her “white” child living as a “poor child,” thereby juxtaposing “white’” and “poor” as opposites:

I am thinking that the child could have been living in a better situation because if people near where I live know about that, they will say this child of a white person is living like a poor child. (Tabarre 2)

Sometimes, the Haitian partners’ acceptance of the peacekeeper-fathered child was explained in relation to the child’s lighter skin phenotype, which was described positively. Proximity to “whiteness” is reflective of colonialisms’ legacy of colorism and shadeism in Haiti wherein lighter skin phenotypes and are sometimes afforded greater social status (Caroline Shenaz Hossein, 2015; Dupuy, 2014; Napoleon, 2021):

But me I did not want any other children which is why I did not have any other child rapidly after him. But actually, I found another man, we got together, and he told me that he loves my child and that he looks like a white man from a foreign country, that he really looks like an actor … I have conceived two other children because he thought that I would have such a beautiful child just like the first child I conceived with the MINUSTAH (Tabarre 3).

Lastly, the degree to which survivors were able to rely on their community and social networks for child rearing support was not only circumscribed by experiences of stigma-related discrimination but also by civil conflict and eroded social networks. For instance, one participant explained that Haiti was in “chaos”:

Well, I had a lot of problems because the country was in chaos; few people were willing to help others. People kept to themselves. So [normally] when you give birth, friends come to help and whatever they have they share with you (Tabarre 2).

## Discussion

Drawing on the perspectives of 18 Haitian women raising peacekeeper-fathered children, we highlighted the interrelated stigma experienced by Haitian mothers and their children. Both the mother and the child were subject to labels: “loose girls” brought into focus the presumed promiscuity and sexual agency of the mothers during the SEA encounter, whereas “child of the MINUSTAH” made visible the child’s paternal origin. The stereotype of impoverishment and adverse living conditions were subsequently attached to both labels. Poverty for the mothers was linked to their perceived socially non-confirming sexual behavior with MINUSTAH peacekeepers, which culminated in paternal abandonment and single motherhood. Consequently, paternal abandonment was understood as being the precipitating factor leading to the experience of child poverty (Vahedi et al., 2020).

Such negative stereotypes, rooted in victim blaming, separated the mothers from other women/girls in the community, including survivors of SEA who did not experience subsequent pregnancy and birth. Furthermore, peacekeeper-fathered children were distinguished from other children in the community on account of being abandoned by their peacekeeper fathers. The process of labeling, stereotyping, and separation culminated in status loss and discrimination through experiences of humiliation, familial abandonment, mothers’ reduced help seeking behaviors and resource utilization, and the children’s threatened learning ability due to relocation and bullying. Thus, the enduring status loss and discrimination experienced by peacekeeper-fathered children and their mothers—as evidenced through intergenerational cycles of poverty and adversity (Lee & Bartels, 2020; Vahedi et al., 2019; Wagner et al., 2020)—results in part through stigmatization, as conceptualized by Link and Phelan (2001). It is important to note that enacted stigma perpetrated by family, community, Haitian men, and SEA survivors without children could also be internalized by the participants. This internalization of stigma could result in feelings of shame, self-blame, or guilt that could limit resource utilization or help seeking (Kennedy & Prock, 2018; Murray et al., 2018).

Further nuancing Link and Phelan’s (2001) conceptualization of stigma is our consideration of stigma actors, spheres of influence, stigma buffers, and the role of the child’s skin phenotype. Stigma was enacted by a variety of actors—family members, Haitian men/stepfathers, SEA survivors without children, and the community at large—across two spheres of influence: public domains where stigma was visible within the community and private domains such as the home and family setting. Buffering the stigma experienced was familial and partner acceptance of the peacekeeper-fathered child and the child’s proximity to “whiteness.” Family members, such as the maternal grandparents and male Haitian romantic partners, sometimes accepted the peacekeeper-fathered child as part of a blended family, thereby reducing stigma enacted in the private sphere. In some cases, acceptance of the peacekeeper-fathered child was described in relation to their light skin phenotype: a form of acceptance reflective of colorism/shadeism and Haiti’s French colonial history. However, such stigma buffers did not necessarily eliminate stigma enacted in the public sphere by other community actors or status loss and discrimination perpetrated by the wider community. Our data did not highlight stigma-by-association among blended family members or the maternal grandparents.

**Figure 1.**
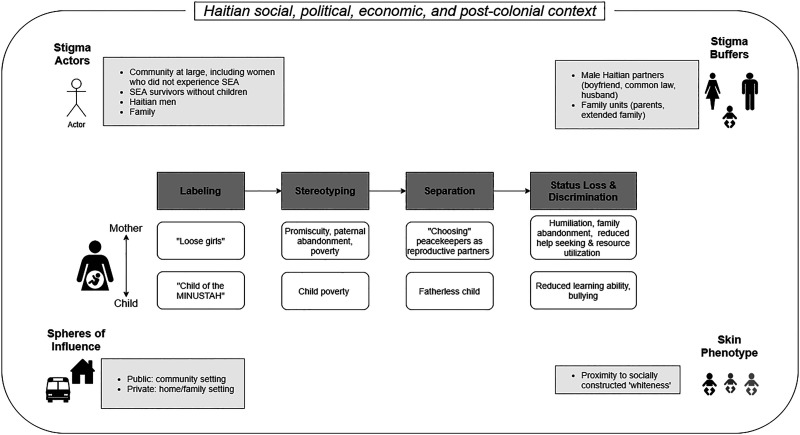
Interrelated Stigma Experiences of Peacekeeper-fathered Children and their mothers in Haiti.

Based on our qualitative data, the lived experiences of stigma are visually summarized in Figure 1, which highlights our application of Link and Phelan’s (2001) conceptualization of stigma to SEA survivors with children, as well as the factors that influence or buffer the process of stigmatization. The stigmatization outlined along with the buffers occur within Haiti’s particular social, political, economic, and post-colonial context. The French colonial context is particularly important when understanding under what conditions “proximity to whiteness” may operate as a stigma buffer (further explored in the discussion). “Whiteness” is a socially constructed identity that refers not to biological race but the expectations and perceived opportunities of “white persons” relative to that of “Black persons,” within a particular socio-political context.

As a form of sexual and gender-based violence, SEA has far reaching social consequences for survivors, particularly when children are conceived. The conception and birth of a peacekeeper-fathered child is a visible mark of the SEA encounter and is analogous to a public disclosure of SEA that is outside of the mother’s locus of control. When peacekeeper-fathered children have lighter skin phenotypes, they are more readily identifiable in public, thereby triggering stigma-related labeling (Wagner forthcoming). In our data, the mothers experienced victim blaming that served to make salient their presumed sexual agency in “enacting” sexual relations with peacekeepers while simultaneously erasing their SEA survivorship and power differential vis a vis the peacekeeper father.

Victim blaming has also been examined in relation to stigmatization and subsequent mental illness among sexual violence survivors in the DRC. Stigmatizing attitudes can take the form of rape myth acceptance, such as believing that survivors of sexual violence are promiscuous, want to be raped, or are getting what they deserve. Schmitt et al. (2021) demonstrated that rape myth acceptance within the social environment was associated with survivor PTSD. Similarly, stigmatization largely explains the mental health impact of sexual violence: depression, PTSD, and hyperarousal (Verelst et al., 2014). Thus, victim blaming sentiments and rape myths are also relevant to the Haitian context and underscore the “loose girl” label ascribed to SEA survivors with peacekeeper-fathered children, thereby initiating the process of stigmatization. This process is similar to stigma experienced by survivors of conflict-related sexual violence in the DRC (Kelly et al., 2012; Schmitt et al., 2021; Verelst et al., 2014; Wachter et al., 2018), however, the identity of the peacekeeper father and the mother–child relationship are further important considerations.

Peacekeeper-fathered children are labeled in relation to the father’s identity. For example, the label “Child of the MINUSTAH” is similar to the colloquial label “child born of the interahamwe” ascribed to children born of genocidal rape in Rwanda (Denov & Piolanti, 2019). Like other CBOW who are subject to stigma on account of their mothers’ relationship with enemy soldiers/rebel groups, peacekeeper-fathered children are connected to the mothers’ history of SEA. However, unlike children born from sexual violence used as a tactic of war, some peacekeeper-fathered children are conceived through long-term transactional sex relationships, wherein the provision of goods and the male provider role obscure exploitative and abusive aspects of transactional relationships (particularly those involving minors) (Mudgway, 2017; Simić & O’Brien, 2014; Vahedi et al., 2019; Vahedi, Lee, et al., 2021). Consequently, mothers of peacekeeper-fathered children are stereotyped as being promiscuous within the context of seeking out sexual relations with “white” or “lighter skin” peacekeepers for the purpose of procreating mixed-race children and achieving upward social mobility.

Given that the child’s lighter skin and mixed race did not improve living conditions, the mothers and children were stereotyped as being impoverished on account of the peacekeepers’ abandonment. Similar themes were noted in a recent study on peacekeeper-fathered children in the DRC, wherein mothers’ perceptions that having a peacekeeper-fathered child would lead to improved social status actually resulted in reduced opportunities and resources for survival (Wagner et al., 2020). Moreover, stigma associated with being “white” or “foreign” amplified the othering of peacekeeper-fathered children in the DRC (Wagner et al., 2020), with a similar pattern occurring in our data from Haiti.

However, we also noted evidence of the child’s proximity to “whiteness” buffering stigma experienced in the private sphere of the home and enacted by family members and stepfathers. In such cases, children were accepted by Haitian romantic partners/stepfathers on account of their physical appearance. Research from the DRC indicated that a husband’s response to the rape of his wife shapes the recovery process and community acceptance (Kelly et al., 2012). In the Haitian context, the male Haitian partner/stepfather’s acceptance might shape experiences of stigma at least in the private sphere. In other cases, the maternal family grew to gradually accept the child irrespective of their proximity to “whiteness”. Such findings are contrary to previous literature suggesting that stepfathers reject children born of war and that raising such children as a single mother prevents affected women/girls from engaging in new partnerships and building families that are in line with social norms (Denov & Piolanti, 2019; Erjavec & Volčič, 2010; Mochmann & Larsen, 2008; Wagner et al., 2020).

The acceptance of peacekeeper-fathered children by the wider blended family may be reflective of Haitian context. Living in blended families may be more socially and culturally acceptable in Haiti where common law partnerships are more common than legal marriages and men openly practice polygamous relationships (Clark, 2006). In addition, shadeism and colorism in Haiti are legacies of French colonial rule and white supremacy. Shadeism and colorism divide women along the lines of skin phenotype (mulatre vs. noir) (Caroline Shenaz Hossein, 2015; Napoleon, 2021). For example, the mixed-race *mulatres* in Haiti comprise a privileged class in Haiti and are distinct from the *blancs* or whites (Caroline Shenaz Hossein, 2015; Dupuy, 2014). However, how this white privilege operates within a context wherein mixed-race peacekeeper-fathered children are abandoned by their fathers and face the realities of poverty has yet to be systematically explored in a post-colonial context. Emerging research illustrates that peacekeeper-fathered children with lighter skin phenotypes experience heightened stigma because the poverty and paternal abandonment they experience runs contrary to the expectation of white privilege and they are subsequently denied the structural advantages of “whiteness” (Wagner fourthcoming). This also has spillover consequences for the mother who is seen as an “active” facilitator of sexual relations with “white” peacekeepers to improve her social standing, and ultimately failed in this regard due to the peacekeeper’s abandonment and impunity.

Given the Haitian history and context surrounding racial hierarchies, mothers raising peacekeeper-fathered children were separated from Haitian women at large and SEA survivors without children. This separation was rooted in the perception that women and girls were willful agents in seeking out sexual relations with “white” or “foreign” UN peacekeepers. In fact, sometimes SEA survivors without children enacted stigma against mothers raising peacekeeper-fathered children: a phenomenon that can be understood as a coping mechanism to reduce their own stigma and elevate their social standing. SEA was perceived as stemming from women and girls’ willful and equal participation in sexual activity. Such behavior runs contrary to norms of female sexual purity. There was little consideration given to the differences between consensual and coerced sexual activity wherein male peacekeepers abused their position of power. In fact the UN largely considers sexual contact between peacekeepers and “beneficiaries of assistance” as SEA due to the power differentials present (United Nations Secretariat, 2003). Recent literature has shed light on how not all sexual relations between host community members and UN peacekeepers are equally exploitative or abusive, with researchers proposing use of an index rather than a binary yes/no categorization for whether SEA is present or not (Gray et al., 2021a; Gray et al., 2021b).

Cross-culturally “good” women and girls are praised for sexual purity, birthing children within the context of a legitimate union, and motherhood (Allman, 1980; Baumeister & Twenge, 2002; Charles, 1995; Ellison, 2003). Such normative understandings of sexual purity are also reflected in Haiti as evidenced though the practice of banm prèv (“give me proof”): the practice of being forced to provide proof of virginity or non-promiscuity in order for sexual violence and related pregnancies to be legitimized (Rahill et al., 2020). Thus, on account of their perceived promiscuity, mothers of peacekeeper-fathered children were perceived as making an active choice to bear children with peacekeepers, regardless of their age at the point of sexual contact, power hierarchies, or the presence of coercion. The peacekeeper’s abandonment was perceived as an inevitable consequence of engaging sexually with peacekeepers and resulted in the peacekeeper-fathered child being separated from other children on account of being fatherless and related to the UN. Similarly, fatherlessness and searching for paternal identity is an enduring theme in the lived experiences of CBOW (Mitreuter et al., 2019; Stelzl-Marx, 2015).

Similar to other literature pertaining to the stigmatization of children born from sexual violence (Bartels et al., 2012) and CBOW (Denov, 2015; Wagner et al., 2020), stigma experienced by SEA survivors and their children culminated in status loss and discrimination. Help seeking avoidance and diminished resource utilization may be a coping mechanism to avoid community enacted stigma, particularly in small and rural towns, thereby illustrating that stigma was internalized by SEA survivors with children. Moreover, any UN-sponsored programs for SEA survivors with peacekeeper-fathered children should critically consider whether victims and their resultant children would be comfortable seeking assistance from the same institution that largely represents the peacekeeper father. Wachter et al. (2018) demonstrated that increased frequency of emotional support seeking was associated with higher symptoms of anxiety and PTSD among women experiencing sexual violence related stigma. Thus, avoidance of help seeking may be perceived as a strategy to ameliorate enacted stigma, even at the detriment of securing needed resources and care. Moreover, the well documented negative effects on educational advancement among CBOW (Mochmann, 2017; Vahedi et al., 2020) were also reflected in our findings: mothers commented on the negative effect of bullying and relocation on their children’s educational prospects.

### Implications

The present findings have implications for the UN and the humanitarian sector. Pursuant to Resolution 62/214: “*Comprehensive strategy on Assistance and Support to Victims of Sexual Exploitation and Abuse by United Nations Staff and Related Personnel*”, the UN has the responsibility and obligation to (i) facilitate, coordinate, and provide material assistance to all complaints and victims as well as (ii) facilitate “the pursuit of claims related to paternity and child support” (United Nations, 2008). The damaging status loss and discrimination enacted through stigmatization is a protection concern for SEA survivors and peacekeeper-fathered children. Accordingly, we argue that stigma should be addressed by the UN pursuant to its responsibilities under Resolution 62/214.

For example, under the obligation that complainants and victims (including peacekeeper-fathered children) receive assistance and support for individual needs arising from the alleged/substantiated SEA, the UN should consider individual level psycho-social supports to ameliorate the internalization of stigma and community outreach to reduce stigmatizing attitudes in public settings such as schools or places of worship. While funded by the UN, such programs should be led, organized, and facilitated by local community organizations, rather than foreign actors. Second, given peacekeeper-fathered children are stigmatized as fatherless children who are embedded in poverty, the facilitation of child support claims and addressing peacekeeper impunity for SEA are key stigma protection concerns. Emerging research from the DRC indicates that peacekeeper-fathered children whose mothers/families maintained a high living standard had higher social status compared to those that experienced poverty (Wagner forthcoming). The UN is situated as a facilitator organization with respect to paternity and child support claims. Thus, we advocate for the UN to invest in establishing sustainable processes and mechanisms to ensure child support payments are provided. Poverty reinforces the stigma experienced by peacekeeper-fathered children and their mothers; thus, the provision of long-term child support is a key protection concern also with respect to stigma.

To date, there is no systematic and longitudinal data on peacekeeper-fathered children. Thus, further research is needed to assess the life course needs of peacekeeper-fathered children across stages of development and settings. For example, research is needed to further understand the effect of gradual familial acceptance as well as colorism/shadeism on mixed-race peacekeeper-fathered children in Haiti and other post-colonial contexts, and how such factors impact stigma enacted by a variety of actors. Further, experiences of stigma-by-association among blended family members of SEA survivors and their peacekeepers should also be explored to better understand the degree to which familial acceptance buffers stigmatization.

### Limitations

The present work has a number of limitations. First, the semi-structured interview guide did not ask specifically about mental health. Thus, we are limited in the ability to connect specific mental health sequela to experiences of stigma, particularly for peacekeeper-fathered children. Second, we used semi-structured interviews to capture lived experiences at one point in time, thus we could not comment on how experiences of stigma change throughout the life course. Further, other methods aligned with the phenomenological tradition include collecting participant journal entries or personal essays; we did not employ such methods of data collection (Smith, 2017). We also limited interviews to mothers of peacekeeper-fathered children; we cannot comment on whether the mothers’ descriptions are a valid representation of stigma experienced by their children. Saturation may also not have been reached in our sample of 18 interviews. For instance, we considered that mothers who were receiving informal financial support from peacekeeper fathers were less motivated to participate in our study and that SEA survivors who choose to abort, abandon, or permanently relocate their peacekeeper-fathered children would not have participated in this research. On account of the interview length (mean time of 23 minutes), participants’ lived experiences may not have been completely described. After consulting with our community partners, we identified a number of factors that contributed to the average length of interview including, interview fatigue, leaving paid work to participate, childcare responsibilities, and the lack of comprehensive probing by the research assistants. Lastly, when translating between English and Kreyòl, subtleties in language and meaning may have been lost.

## Conclusion

This research explored the lived experiences of stigma among Haitian SEA survivors and their peacekeeper-fathered children. We applied Link and Phelan’s (2001) conceptualization of stigma to identify and describe the processes of labeling, stereotyping, separation, as well as status loss and discrimination. Stigmatization culminated in reduced help seeking behavior and resource utilization among the mothers and inhibited educational advancement for the children. Potential factors buffering the stigma experienced include gradual acceptance by the maternal family and stepfather and the child’s proximity to “whiteness.” We advocate that the UN address stigma as a protection concern, pursuant to its responsibilities as outlined by Resolution 62/214.
